# Diagnosis of mycobacterial infections based on acid-fast bacilli test and bacterial growth time and implications on treatment and disease outcome

**DOI:** 10.1186/s12879-016-1474-6

**Published:** 2016-04-01

**Authors:** Fabiane N. Riello, Rebecca T. S. Brígido, Sergio Araújo, Tomaz A. Moreira, Luiz Ricardo Goulart, Isabela M. B. Goulart

**Affiliations:** National Reference Center for Sanitary Dermatology and Leprosy (CREDESH) Clinical Hospital Federal University of Uberlândia, Uberlândia, Minas Gerais Brazil; Laboratory of Clinical Analysis, Clinics’ Hospital of the Federal University of Uberlândia, Uberlândia, Minas Gerais Brazil; Federal University of Uberlandia, Institute of Genetics and Biochemistry, Laboratory of Nanobiotechnology, Campus Umuarama, Block 2E, Room 248, CEP 38400-902 Uberlandia, Minas Gerais Brazil

**Keywords:** Mycobacteria, PCR-RFLP, Molecular Diagnosis

## Abstract

**Background:**

The establishment of therapeutic regimens for mycobacteriosis depends on the accurate identification of *Mycobacterium* species, and misdiagnosis can result in inappropriate treatment and increased mortality of patients. Differential diagnosis among *Mycobacterium* species has been made by conventional phenotypic and biochemical tests after a long culture period. Specialized molecular diagnostics of mycobacteria allows rapid detection and species identification; however, such tests are not available in public health programs. Our aim was to demonstrate the clinical implications of erroneous diagnosis by performing molecular genotyping of mycobacterial infections in patients that were diagnosed based on symptoms, culture and bacilloscopy.

**Methods:**

Culture samples of mycobacterial infections from 55 patients clinically diagnosed as tuberculosis in 2013 and 2014, based on conventional methods, were identified by PCR -RFLP and results are discussed.

**Results:**

We have confirmed 35 (63.6 %) positive samples as *M. tuberculosis*, but 18 (32.7 %) were identified as non-tuberculous mycobacteria (*M. avium* type 1, *M. avium* type 2, *M. kansasii* type 1 type 1, *M. mucogenicum*, *M. chelonae*, M. terrae type 3, and 1 unknown RFLP pattern) and two were negative. Regarding clinical diagnosis, 61.8 % (34/55) was classified as pulmonary tuberculosis. It is important to emphasize that 36.4 % (20/55) of samples were misdiagnosed by conventional methods, and 11 (61.1 %) of the HIV positive patients (18/55) were NTM-coinfected.

**Conclusion:**

The identification of species in mycobacterial infections is essential for correct diagnosis and choice of treatment regimen, and misdiagnosis by conventional tools can lead to chronic disease, increased resistance and death.

**Electronic supplementary material:**

The online version of this article (doi:10.1186/s12879-016-1474-6) contains supplementary material, which is available to authorized users.

## Background

Despite the large number of *Mycobacterium* species [1], *M. tuberculosis* (MTB) is still one of the major causes of human diseases and mortality worldwide. The proportion of mycobacterial infections due to nontuberculous mycobacteria (NTM) seems to be increasing in relation to TB infections, probably due to improved diagnosis through molecular analysis, but also due to increasing incidence of immunocompromised patients with HIV infection [2, 3], chronic inflammatory diseases [4], organ-transplantation [5], and dermatological diseases [6], leading to significant NTM-related deaths in individuals older than 55 years of age [7]. In Brazil, NTM infections have become an epidemiological emergency, especially after the outbreak of rapidly growing mycobacteria in post-surgical infections from 2003 to 2009 [8]. All these variable co-morbidities together with factors that remain unknown pose great challenges to the epidemiological analysis of NTM diseases and application of proper treatments, greatly due to their misdiagnosis.

Differentiation between mycobacteria species is typically made in positive cultures based on phenotypic and biochemical traits. Currently, the diagnostic methods used are bacilloscopy and microbiological culture [9], but the main method for bacilli detection is the Ziehl-Neelsen specific staining technique; however, despite its simplicity and low cost, it has a very low sensitivity (30 to 50 % of the *M. tuberculosis* cases are negative). The microbiological culture is generally used in suspected pulmonary cases and in negative bacilloscopy, and allows detection and isolation of mycobacteria for subsequent identification of the isolated complex [9]. Despite its importance, the *M. tuberculosis* culture is time consuming due to its slow growth, and not always presents 100 % positivity [10]. Automated detection systems of mycobacteria, such as the BACTEC 460 TB®, BACTEC 9000®, and the MGIT® are promising, but they can also produce false-positive results due to contamination by other bacteria [9].

In places without appropriate identification techniques, the NTM diseases can be confounded with tuberculosis due to the similarity of clinical symptoms, affected sites and morphological characteristics of bacilli, leading to misdiagnosis and improper treatment, resulting in prolonged treatment, chronic infections, drug resistance and increased mortality [11].

The establishment of therapeutic regimens depends on species identification of mycobacteria, which can be critical for the adoption of appropriate therapy [12]. The mycobacteriosis treatment becomes even more complex due to increasing resistance and low sensitivity to tuberculostatic drugs. Therefore, for an efficient and proper treatment, the identification of the causative agent and its drug sensitivity are necessary in order to choose appropriate drugs [13].

Tools commonly used for diagnosis of tuberculosis in resource-limited settings, such as the direct Ziehl-Neelsen (DZN) staining and the direct fluorescent microscopy (DFM) have been compared, which have missed 64 and 20 % of cases, respectively, supporting the concern that using DZN alone may risk missing the majority of TB cases [14]. The need for fast and reliable laboratory tests for mycobacterial diagnosis resulted in the development of molecular methods for detection and identification of species directly from clinical specimens and/or from isolated culture colonies. However, while some techniques are simple, others have complex requirements, and the incorporation of these tests within a country's national health program must be carefully determined [15]. It is important to consider that increasing antibiotic resistance of pathogens associated with nosocomial infections and laboratorial contamination also pose major challenges to health programs, especially in regions with poor settings, for which no single measures can solve this complex problem. Developed nations must strategically aid those regions to prevent the increased burden of antibiotic-resistant mycobacteria due to the lack of proper diagnosis and patients’ management.

Several molecular methods have been developed for the identification of mycobacteria species [16]. Among them, the PCR-RFLP (Restriction Fragment Length Polymorphism)-based method, despite its moderate complexity, was shown to be fast, with good accuracy, and cost-effective [17]. The method, also referred as PRA-hsp65, amplifies and digests a portion of the hsp65 (65 kDa heat shock protein) with restriction endonucleases, and is able to detect the most important and common mycobacteria species. This method has the advantages of having high specificity, rapid results, and requires only two equipments; a thermocycler and agarose gel electrophoresis [18].

Another technique that has been used to differentiate mycobacteria species, and is gradually replacing the conventional PCR, is the real-time PCR, which has the advantages of releasing results in reduced time with minimal manipulation, and higher sensitivity. However, the this technique has some impediments, such as high cost, besides not being able to identify many species in a single reaction, frequently producing false-positive results hindering the lack of standardization of this technique [16]. Thus, even with the most advanced techniques, PCR-RFLP continues to be the most useful tool for the identification of mycobacteria species.

This study aimed to identify species of mycobacteria present in samples of positive cultures from patients diagnosed with tuberculosis treated at the Clinical Hospital of the Federal University of Uberlândia in the biennium 2013-2014, and discuss the implications of misdiagnosis by using only conventional bacilloscopy and culture methods.

## Methods

### Study design and patients

A transversal retrospective descriptive study was performed in culture samples from all patients diagnosed with tuberculosis and treated at the Clinics’ Hospital of the Federal University of Uberlândia during the biennium 2013-2014. Age, sex, HIV status, and patient treatment time were obtained in their medical records. Two or more cultures were performed for each patient for clinical evaluation, and only those positive cultures were subsequently used for DNA extraction and analyses.

### Sampling

Among 1380 cultures of suspected patients with mycobacterial infections, fifty-five clinical samples (bronchial aspirate, cervical lymph nodes, bone marrow aspirate, sputum, pleural liquid, vesicular fluid, bronchial lavage, tracheal secretion, gastric aspirate, liquor, gingival injury, surgical scar and liver fragment) with positive and viable cultures of *Mycobacterium sp*. were collected, which were clinically diagnosed as tuberculosis. Important to emphasize that the sample size does not represent the entire suspected population. Negative culture samples were not investigated by PCR, which is a limitation of this study.

Fifteen reference samples of mycobacterial species were used for molecular standard patterns, which were previously identified and donated by the Oswaldo Cruz Foundation (FIOCRUZ/RJ): *M. avium, M. abscessus, M. fortuitum, M. terrae, M. peregrinum, M. smegmatis, M. marinum, M. phlei, M. gordonae, M. kansasii, M. bovis, M. szulgai, M. massiliense*. The *M. tuberculosis* reference strain was provided by the Instituto Hélio Fraga (Rio de Janeiro, Brazil), and the *M. leprae* reference strain was provided by the National Reference Center of Sanitary Dermatology and Leprosy (CREDESH/HC/UFU, Uberlandia, MG, Brazil).

### Ethical statement

Culture samples from various tissues and body fluids of patients with clinical diagnosis of tuberculosis from the Infectious Diseases Ambulatory Service of the Clinics’ Hospital of UFU were obtained for molecular genotyping. Informed Consents were obtained from all patients, who were invited to participate in the study approved by the Research Ethics Committee of the Federal University of Uberlândia, under the process numbers CEP/UFU N. 123/10 (Approval CEP 457/10, Tuberculosis) and CEP/UFU N. 355/11 (Approval CEP 097/12, HIV and co-infections). Informed consents from two patients under 16 years of age were also obtained from their parents.

### Bacilloscopy and culture

Bacilloscopy and mycobacteria culture were performed by the Laboratory of Clinical Pathology of the Federal University of Uberlândia, according to the recommendations outlined in the National Manual of Tuberculosis Surveillance and Other Mycobacteria [19]. Clinical symptoms that strongly indicate the presence of mycobacteria and/or positive bacilloscopy for acid-fast bacillus resistant (AFB) are criteria for confirmatory culture of patients’ samples. Duplicate cultures of different clinical specimens were performed on Middlebrook 7H9 broth medium for growth, storage, and subsequent DNA extraction. Results regarding culture time and bacilloscopy were recorded.

### DNA extraction

Positive cultures in the Middlebrook medium were heat-inactivated at 95 °C for 15 min and processed in three replicates. Subsequently, DNA extraction was performed by the salting-out method [20].

### DNA amplification and restriction endonuclease digestion (PCR-RFLP)

The 439-bp fragment of the Hsp65 gene was amplified using the Tb11 (5'-ACCAACGATGGTGTGTCCAT-3’) and Tb12 (5′-CTTGTCGAACCGCATACCCT-3′) primers modified from the protocol described elsewhere [18]. The PCR reaction components for a final volume of 50 μL consisted of: 25 μL of 2X PCR Master Mix [0.05 U/μL Taq DNA polymerase, reaction buffer, 4 mM MgCl_2_, 0.4 mM of each dNTP (dATP, dCTP, dGTP and dTTP) (Thermo Scientific), 1.5 μL of 10 μM of each primer, 4 μL of sample DNA (25 ng/μL) and 18 μL of ultrapure water. The PCR reaction consisted of 45 cycles with the following conditions: 1 min at 94 °C, 1 min at 60 °C, 1 min at 72 °C, followed by a final extension of 10 min at 72 °C.

For endonucleases’ restrictions with BstEII and HaeIII, 15 μL of the PCR product was added to a mix containing 0.5 μL (5U) of the enzyme, 2.5 μL of restriction buffer (5X buffer B), 0.2 μL of BSA and 11.3 μL of ultrapure water. The BstEII reaction was incubated for 60 min at 60 °C and the HaeIII reaction was performed at 37 °C for 3 h.

After restriction, 8 μL of digested products were added to 3 μL of running buffer of agarose gel (0.25 % bromophenol blue, 40 % sucrose in water) and separated by 3 % agarose gel electrophoresis in 0.5X TBE buffer. Fragments were visualized under UV light using ethidium bromide as a fluorescent DNA intercalator. A 25-bp molecular marker was used for fragment sizing. Electrophoresis banding patterns were submitted to algorithms described in the online tool PRASITE (http://app.chuv.ch/prasite/index.html) for species identification.

### Diagnosis

Clinical diagnosis of mycobacterial infections performed at the Clinics’ Hospital of Uberlândia is based on clinical symptoms, bacilloscopy and bacterial growth time, and the final interpretation is made by physicians, following the standard protocol of the Brazilian Ministry of Health [19]. Clinical diagnosis of tuberculosis is reached when patients present clinical symptoms, positive bacilloscopy and/or positive slow-growing culture for *Mycobacterium sp*. All positive cultures that were clinically diagnosed as TB were submitted to molecular identification of mycobacteria species. Misdiagnosis was considered when the PCR-RFLP identified other mycobacteria species that were different from *M. tuberculosis*.

### Treatment

Since patients with *Mycobacterium sp*. were initially diagnosed with tuberculosis, the first line of treatment chosen was the standard regimen recommended by the Brazilian Ministry of Health (2RHZE/4RH): two months of COCXIP 4 (rifampicin, isoniazid, pyrazinamide and ethambutol), and four more months of rifampicin and isoniazid. Substitute treatments with other complementary drugs were initiated when treatment failed, probably due to misdiagnosis or bacterial resistance. Medications commonly used in suspected cases or with confirmed infection of atypical mycobacteria are: clarithromycin, ciprofloxacin, amikacin, cefoxitin, imipenem, doxycyclin or azithromycin.

## Results

### Patients’ data

In the period of 2013 to 2014, 55 positive mycobacterial cultures were obtained from patients diagnosed with tuberculosis attended at the Clinics’ Hospital of the Federal University of Uberlândia. Considering the demographical characteristics, 40 patients were male (72.7 %), with an average age of 46 years old, ranging from 11 to 92 years (Table 1).

**Table 1:** Characterization of NTM-infected patients diagnosed with tuberculosis based on conventional procedures compared with molecular methods

Patient ID	Age	HIV Status	Sample type	AFB	Culture/Period	Clinical Diagnosis	PCR-RFLP	Treatment	Clinical Outcome
9	67	-	Sputum	+	Myc.sp/24 days	Pulmonary Tuberculosis	*M. avium* type 1	Substitute treatment	Under treatment
10	23	+	Gastric Aspirate	+	Myc.sp/25 days	Tuberculosis unspecified	*M. avium* type 1	Standard treatment	Died
11	65	+	Bronchial Aspirate	-	Myc.sp/30 days	Pulmonary Tuberculosis	*M. avium* type 1	Substitute treatment	Under treatment
14	83	-	Bronchial Aspirate	+	Myc. sp/no info.	Pulmonary Tuberculosis	*M. kansasii* type1	Standard treatment	No adherence to treatment
21	45	+	Bronchial Aspirate	-	Negative	Pulmonary Tuberculosis	Negative	Standard treatment	Completed treatment
22	77	-	Sputum	-	Myc. sp/>15 days	Tuberculosis unspecified	*M. terrae* type 3	Substitute treatment	No adherence to treatment
25	83	-	Bronchial Aspirate	-	Myc. sp/30 days	Pulmonary Tuberculosis	*M. intracellulare* type 1	Standard treatment	Completed treatment
28	22	+	Liquor	-	Myc. sp./no info.	Meningitis - Tuberculosis	*M. avium* type 2	Standard treatment	Under treatment
31	57	+	Liquor	-	Myc. sp./no info.	Pulmonary Tuberculosis	Negative	Substitute treatment	Completed treatment
32	52	+	Bronchial Aspirate	-	Myc. sp/45 days	Pulmonary Tuberculosis	*M. avium* type 1	Standard treatment	Died
33	36	+	Gastric Aspirate	-	Myc. sp/20 days	Miliary Tuberculosis	*M. avium* type 2	Substitute treatment	Died
34	92	-	Bronchial Aspirate	-	Myc. sp/18 days	Pulmonary Tuberculosis	NTM unknown pattern	Standard treatment	No adherence to treatment
36	52	-	Bronchial Aspirate	+	Myc. sp/>15 days	Pulmonary Tuberculosis	*M. kansasii* type1	Standard treatment	Under treatment
37	24	+	Bronchial Aspirate	+	Myc. sp/35 days	Pulmonary Tuberculosis	*M. avium* type 1	Standard treatment	Completed treatment
38	42	+	Liquor	-	Myc. sp/16 days	Bone Tuberculosis	*M. avium* type 1	Standard treatment	Completed treatment
39	40	+	Cervical Lymph node	-	Myc. sp/45 days	Lymph Node Tuberculosis	*M. avium* type 1	Standard treatment	Completed treatment
40	29	-	Bronchial Aspirate	-	Myc. sp/10 days	Pulmonary Tuberculosis	*M. mucogenicum*	Standard treatment	No adherence to treatment
41	44	+	Bone marrow Aspirate	-	Myc. sp >20 days	Miliary Tuberculosis	*M. avium* type 1	Standard treatment	Completed treatment
52	59	+	Gastric Aspirate	-	Myc. sp/25 days	Miliary Tuberculosis	*M. avium* type 1	Standard treatment	Under treatment
54	77	-	Surgical Scar	-	Myc. sp/05 days	Bone Tuberculosis	*M. chelonae*	Substitute treatment	Under treatment

### Microbiological data

**Fig. 1:**
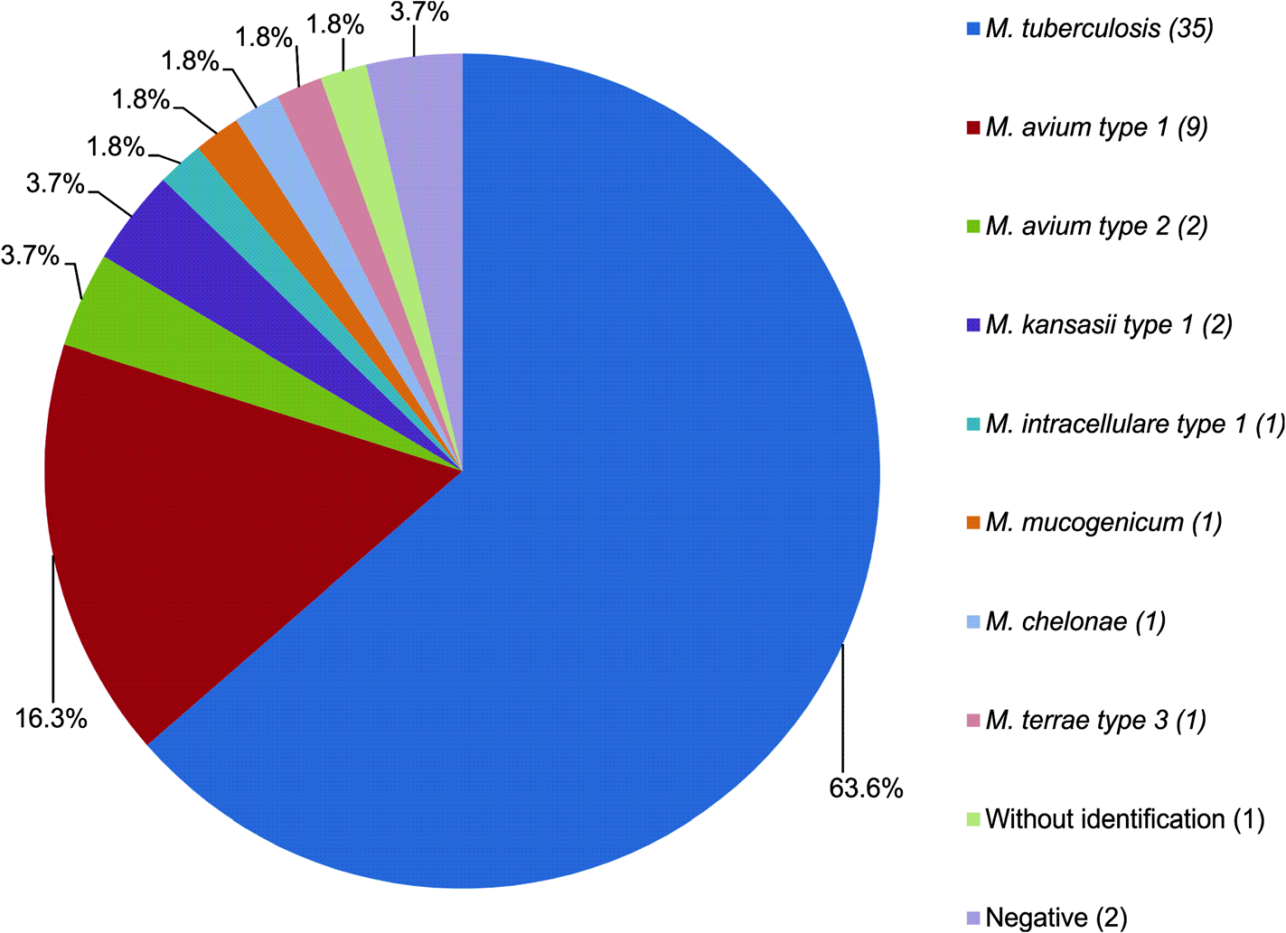
Frequency distribution of mycobacteria species identified by PCR-RFLP in patients clinically diagnosed with tuberculosis

The observed frequency of mycobacterial species were: 63.6 % (35/55) of *M. tuberculosis*, 16.3 % (9/55) of *M. avium* type 1, 3.7 % (2/55) of *M. avium* type 2, 3.7 % (2/55) of *M. kansasii* type 1, 1.8 % (1/55) of *M. intracellulare* type 1, 1.8 % (1/55) of *M. mucogenicum*, 1.8 % (1/55) of *M. chelonae*, 1.8 % (1/55) of *M. terrae* type 3, and 1.8 % (1/55) presented an unknown pattern (HaeIII-150/110; BstEII-235/210). Two patients diagnosed with TB clinical symptoms (3.7 %) were both AFB and PCR-RFLP negatives (Tables 1 and 2, and Fig. 1), probably due to misdiagnosis, although one of them presented a positive culture with an unknown microorganism. Co-infections by more than one mycobacteria species in the same sample were not identified, since mixed RFLP patterns from primary cultures were not observed. It is important to emphasize that DNA extraction was performed in primary liquid cultures (pools) and not from a single colony.

**Table 2:** Patients’ characterization according to the molecular identification of mycobacteria species

Patients’ Characterization		Molecular Diagnosis (PCR-RFLP)										
		*M. tuberculosis*	*M. avium* type 1	*M. avium* type 2	*M. kansasii*	*M. intracellulare* type 1	*M. mucogenicum*	*M. chelonae*	*M. terrae* type 3	MNT unknown	Negative	Total
Gender	Male	26	6	2	2	1	0	1	0	0	2	40
	Female	9	3	0	0	0	1	0	1	1	0	15
Age	<25 years	4	2	1	0	0	0	0	0	0	0	7
	25-50 years	17	3	1	0	0	1	0	0	0	1	23
	>50 years	14	4	0	2	1	0	1	1	1	1	25
AFB	Positive	21	3	0	2	1	0	0	0	0	0	27
	Negative	14	6	2	0	0	1	1	1	1	2	28
Culture time	>15 days	30	9	1	1	1	0	0	1	1	0	44
	<15 days	5	0	0	0	0	1	1	0	0	0	7
	Without information	0	0	1	1	0	0	0	0	0	2	4
Clinical diagnoses	Pulmonary tuberculosis	23	4	0	2	1	1	0	0	1	2	34
	Unspecified tuberculosis	3	1	0	0	0	0	0	1	0	0	5
	Miliary tuberculosis	3	2	1	0	0	0	0	0	0	0	6
	Pleural tuberculosis	3	0	0	0	0	0	0	0	0	0	3
	Lymph Node tuberculosis	1	1	0	0	0	0	0	0	0	0	2
	Bone tuberculosis	0	1	0	0	0	0	1	0	0	0	2
	Vesical tuberculosis	1	0	0	0	0	0	0	0	0	0	1
	Meningitis - tuberculosis	1	0	1	0	0	0	0	0	0	0	2
HIV Status	HIV positive	6	8	2	0	0	0	0	0	0	2	18
	HIV negative	29	1	0	2	1	1	1	1	1	0	37

Among 35 *M. tuberculosis* samples identified by PCR-RFLP, 21 were AFB positive. For the positive NTM samples (18/55; 32.7 %), 5 were AFB positives (3 *M. avium* type 1 and 2 *M. kansasii*) and 13 were AFB negatives (6 *M. avium* type 1, 2 *M. avium* type 2, 1 *M. terrae* type 3, 1 *M. Intracelulare,* 1 *M. mucogenicum*, 1 *M. chelonae*, 1 NTM with unknown pattern). The two remaining samples were both PCR and AFB negatives (Tables 1 and 2).

### Culture vrs PCR-RFLP

Among the 35 *M. tuberculosis* cultures identified by PCR-RFLP, 30 presented slow growth (>15 days) and 5 had fast growth (<15 days). From the 9 *M. avium* type 1 cultures, all had growth periods greater than 15 days. From the two *M. kansasii* type 1cultures, one had 15-day growth and one had no information about culture time. From the two *M. avium* type 2 cultures, one presented growth in more than 15 days and the other had no information. The *M. chelonae* and *M. mucogenicum* cultures had growth times lower than 10 days. The *M. terrae* type 3 culture grew in 15 days. The NTM species with unknown patterns had growth periods of more than 15 days. From the two negative cultures that were molecularly detected and typed, one had no information about its culture time and the other grew in 15 days (Tables 1 and 2).

### Affected sites

Among TB clinically diagnosed samples, 61.9 % (34/55) were found in the pulmonary site, in which 23 were *M. tuberculosis*, four *M. avium* 1, two *M. kansasii*, one *M. intracellulare*, one *M. mucogenicum*, one unknown NTM and two were negative. Five samples involved unspecified sites (9 %; 5/55), which were classified as *M. tuberculosis* (3), *M. avium* 1 (1) and *M. terrae* (1). Disseminated infection (miliary tuberculosis) was reported in 11 % (6/55) of the positive samples, including *3 M. tuberculosis*, 2 *M. avium* 1 and 1 *M. avium* 2. The 5.5 % (3/55) of positive pleural sites were infected by *M. tuberculosis*.

Among the extra-pulmonary tuberculosis, 3.6 % (2/55) were ganglionar (1 *M. tuberculosis* and 1 *M. avium1*), 3.6 % (2/55) were bone-derived (1 *M. chelonae* and *M.avium1*); 1.8 % (1/55) was vesical (*M. tuberculosis*); and 3.6 % (2/55) presented affected meninges and liquor (*M. tuberculosis* and *M. avium* 2) (Table 2).

### HIV Co-infection

Regarding HIV co-infections, we have found that 32.7 % (18/55) of the patients were HIV positive and among them, 44.4 % (8/18) were *M. avium* type 1, 33.3 % (6/18) were *M. tuberculosis*, 11.1 % (2/18) *M. avium* type 2, and 11.1 % (2/18) were PCR negative (Table 2). Therefore, 56 % of co-infections with HIV were caused by NTM.

### Misdiagnosis and inappropriate treatment

All patients were clinically diagnosed with tuberculosis, but only 63.6 % (35/55) were confirmed as *M. tuberculosis* by PCR-RFLP; therefore, 36.4 % (20/55) of the patients were misdiagnosed. Among them, 18 samples were identified as NTM infections, and 2 were negatives (Tables 1 and 2).

Concerning the treatment, 89 % (49/55) of patients were submitted to the standard treatment for tuberculosis [2RHZE/4R - two months of COCXIP 4 (rifampicin, isoniazid, pyrazinamide and ethambutol) followed by four additional months with rifampicin and isoniazid], and only 11 % (6/55) had a substitute treatment for atypical mycobacteria, which included auxiliary drugs to the standard treatment, due to the chronic infection. Among those 6 chronic patients, 5 (7.3 %) were NTM-infected and had their treatment replaced by the appropriate treatment, and one patient was negative (Tables 1 and 2). Therefore, 13 out of 18 NTM-identified were inappropriately treated as tuberculosis.

## Discussion

Nontuberculous mycobacteria infections have increased considerably in Brazil and worldwide, probably due to the reduced incidence of tuberculosis as a consequence of treatment improvements, which in turn make individuals, especially immunosuppressed ones, more vulnerable to opportunistic mycobacteria. Although this fact was first evidenced in developed countries, it has been increasingly observed in developing countries [21]. This augment can also be justified by the deployment of new techniques for species identification, which have improved the detection of NTM species, contributing to a better understanding of the epidemiology of mycobacterial infections.

In Brazil, there are few studies about nontuberculous mycobacteria epidemiology [9, 17, 22], because NTM infections have no obligatory notification, and besides none of the public hospitals and health services perform molecular methods for species identification. Also, these reports are isolated studies, mainly in the Southeastern states, with different methodologies, unavailable in most of the Brazilian states [22], so comparisons among them are difficult. The majority of investigations present restricted species identification of NTM, and only two reports associate with epidemiological data, infected body parts, diagnosis and treatment [17, 23]. In Uberlândia, a city with high flow of migrants and rapid development in central Brazil, there are still no records of mycobacterial infections.

This study performed in a reference hospital of Uberlandia, Minas Gerais State, Brazil, identified mycobacteria species by PCR-RFLP, and compared results of patients’ samples that were clinically diagnosed as tuberculosis infection, according to a conventional method algorithm using clinical symptoms, bacilloscopy and culture. We opted for the PCR-RFLP method due to its rapidity, low cost, reproducibility, and capability of identifying a large number of NTM species [17, 24]. Another reason was to evaluate its effectiveness for routine implementation in the Brazilian unified health system (SUS). Here, we showed a significant percentage of misdiagnosis (36.4 %), which has led to inappropriate treatment of 13 out of 18 patients (72.2 %), demonstrating the importance of species identification.

When we characterized the patient profiles with mycobacteriosis, we found that about 70 % were male and the mean age was 46 years, which is corroborated by a report elsewhere [25] that found that the majority of affected patients was male (76 %) with an average age of 40 years old. This may be partially explained by the predominance of middle-age men migrants that come from low-income regions looking for better opportunities.

The identification of *M. tuberculosis* infections in 63.6 % (35/55) of samples is consistent with other studies that found a higher prevalence of MTB; 67 % [26], 57.5 % [27], 78.6 % [28] and 77 % [29]. Despite the higher occurrence of MTB, it has been shown elsewhere that the MTB frequency is declining over the years, while the NTM frequency is significantly increased, with prevalence ranging from 9.1 % in 2003 to 31.9 % in 2009 [28]. The increased NTM prevalence over time is due to the improvement of techniques for identification and to better tuberculosis diagnosis and treatment, and could also be partially attributed to the increase in immunosuppressed people, with HIV, organ transplantation, and other causes.

In most studies similar to this, the NTM complex most commonly associated with the clinical disease were *M. avium, M. fortuitum, M.kansasii, M. chelonae, M. gordonae* and *M. abscessus* [17]. In this study, among the potentially pathogenic NTM species, we observed 50 % (9/18) *M.avium* type 1, 11.1 % *M. avium* type 2, 5.5 % *M. intracellulare*, *M. kansasii* type 1 (11.1 %) and *M. chelonae* (5.5 %). We also found two rarely pathogenic species, such as 1 *M. mucogenicum* (5.5 %) and 1 *M. terrae* type 3 (5.5 %), although they are not commonly associated with diseases they are found in the environment and in potable water distribution systems due to its resistance potential in disinfection procedures [21]. We also identified a NTM sample with unknown pattern, for which DNA sequencing will be performed to determine the existence of a new species.

Regarding the mycobacteria culture, it is important to note that despite the slow growth of *M. tuberculosis* (2-8 weeks), 5 MTB cultures presented fast growth (<15 days) (Additional file 1: Table S1), probably due to the high inoculum concentration, which may have also interfered in the clinical diagnosis. These five cases were correctly treated as MTB, because all cases are initially submitted to TB treatment regimen; however, because of the lack of molecular analysis, the treatment could only be continued as NTM infection if it had failed, and this may have had an important clinical impact on NTM cases that may lead to chronic disease, but fortunately this was not the case.

The bacilloscopy is not a reliable method too, since it can be negative in 30 to 50 % of cases of individuals infected with *M. tuberculosis* [9]. In this study, we found that 38 % of the MTB positive samples were AFB negative, whereas 72 % of the NTM infections were AFB negative.

It is common to find high rates of immunosuppressed individuals affected by NTM species, especially the *M. avium* complex [30]. We found 55 % (10/18) of HIV positives co-infected with *M avium* type 1 and 2 species. However, it is interesting to highlight that 45 % of NTM infections occurred in supposed immunocompetent individuals. Epidemiologists admit that for this type of patient it is important to recognize other risk factors associated with higher chances of developing NTM, such as pre-existing pulmonary lesions or chronic diseases that cause major structural damage, predisposing the individual to the development of NTM pulmonary disease [31, 32].

It is important to emphasize that NTM positivity in clinical samples must be carefully interpreted, and may have three meanings: (a) the mycobacterium is the etiological agent, in which diagnosis is supported by clinical signs and by repeated isolation of this bacillus in the same patient, or by a single isolation in the case of samples taken aseptically; (b) the mycobacterium may have colonized the sample, but has no clinical significance, which may have occurred due to the use of contaminated equipment, a common phenomenon called “pseudo-infection”; or (c) the detection of mycobacteria in clinical samples comes from laboratory contamination (contaminated solutions), which can be easily verified with proper controls or by observing if the same mycobacteria was isolated from other samples analyzed in the same day [24].

The diagnosis performed at the Clinics’ Hospital of Uberlândia, takes into account only bacilloscopy, culture time and symptoms for clinical diagnosis, and among 55 patients diagnosed as tuberculosis, 35 were correctly identified as MTB (63.6 %) when verified by PCR-RFLP, but 36.4 % (20/55) of these samples were either NTM-infected (18) or negative (2), configuring misdiagnosis. The disease caused by NTM in most cases is pulmonary or disseminated [33], but our findings, demonstrated that the largest number of pulmonary disease (51 %; 18/35) occurred primarily in tuberculosis patients, while for the NTM, 39 % (7/18) were pulmonary, and 16.6 % (3/18) were miliary disease. Misdiagnosis of this group of patients may have been the cause of multiple sample collections, and patients’ physical, psychological, economic and social suffering due to the inadequate treatment. As an additional consequence, NTM strains may have become antibiotic resistant, prolonging the treatment, and in most cases resulting in chronic disease, leading to many hospitalizations and unsuccessful treatments, and increasing the chances of liver damage and mortality [9].

The recommended treatment for MTB applied to NTM-infected patients, or for NTM applied to MTB patients, or for NTM with non-specific schemes may lead to major complications, since different therapeutic regimens should be adopted for each group. The antimicrobial and the treatment time varies according to the species, and some NTM are resistant to the drugs used for tuberculosis treatment, and not all NTM are sensitive to the same treatments, which may result in chronic infections [12]. Available studies consider the treatment of the NTM disease quite complex, and recommend the observation of three factors to guide the therapeutic decision: the bacillary load, the isolated species and the presence of clinical progression of the disease. The establishment of appropriate treatment regimens may be complicated by the large number of NTM species with differential antimicrobial susceptibility profiles. Currently, at international level, treatment recommendations for NTM infections are based on a series of case reports that consider the treatment experiences of the *M. avium* and *M. kansasii* complex, which constitute most of the cases described in the literature [34, 35].

## Conclusion

Briefly, we have demonstrated that conventional methods used to detect mycobacterial infections, AFB and culture, have led to significant uncertain diagnoses, and considering that species identification is essential to choose the correct therapeutic regimen, it is expected then that mycobacteria resistance, chronic infections, and poorer outcomes may be direct consequences of such misdiagnosis. We suggest that urgent molecular identification of mycobacteria species and resistance tests are incorporated into public health systems in regions with resource-poor settings in order to reduce morbidity by adopting appropriate therapeutic regimens, and to prevent the increased burden of antibiotic-resistant mycobacteria.

### Availability of data and materials

All the data supporting our findings are contained within this work.

## Additional file

Additional file 1: Table S1. Characterization of MTB-infected patients diagnosed with tuberculosis based on conventional procedures compared with molecular methods. (DOC 84 kb)

## Abbreviations

AFB: Acid-Fast Bacilli
BstEII and HaeIII: restriction enzymes (endonucleases)
CREDESH: National Reference Center of Sanitary Dermatology and Leprosy
Coxcip 4: rifampicin, isoniazid, pyrazinamide and ethambutol
dATP: Deoxyadenosine triphosphate
DFM: Direct fluorescent microscopy
dCTP: Deoxycytidine triphosphate
dGTP: Deoxyguanosine triphosphate
dNTP: Deoxynucleotide triphosphates
dTTP: Deoxythymidine triphosphate
DZN: Direct Ziehl-Neelsen
FIOCRUZ: Oswaldo Cruz Foundation
HC/UFU: Clinical Hospital Federal University of Uberlândia
HIV: Human Immunodeficiency Virus
Hsp65: 65 kDa heat shock protein
min: Minutes
MTB: *Mycobacterium tuberculosis*
NTM: Nontuberculous mycobacteria
PCR: Polymerase chain reaction
PRA: Restriction Enzyme Analysis
PRASITE: site (http://app.chuv.ch/prasite/index.html)
RFLP: Restriction Fragment Length Polymorphism
SUS: Brazilian unified health system
U: Unit
μL: Microliter
mM: Milimolar
μM: Micromolar
°C: Degrees Celsius
2RHZE/4RH: two months rifampicin, isoniazid, pyrazinamide and ethambutol/four months rifampicin and isoniazid
