# Supplementary material for: Diagnosis of mycobacterial infections based on acid-fast bacilli test and bacterial growth time and implications on treatment and disease outcome
Source: BMC Infect Dis. 2016 Apr 1;16:142. doi: 10.1186/s12879-016-1474-6 (PMC4818481; doi:10.1186/s12879-016-1474-6)
Supplement: Additional file 1: Table S1. — Characterization of MTB-infected patients diagnosed with tuberculosis based on conventional procedures compared with molecular methods. (DOC 84 kb) [file 12879_2016_1474_MOESM1_ESM.doc]

**Table S1.**Characterization of MTB-infected patients diagnosed with tuberculosis based on conventional procedures compared with molecular methods.

Myc. sp= mycobacterium without mention of specific species; Fem = Female; Male = Male.

| **Patient ID** | **Age** | **HIV**  **Status** | | **Sample type** | **AFB** | **Culture/**  **Period** | **Clinical Diagnosis** | **PCR-RFLP** | **Treatment** | **Clinical Outcome** |
| --- | --- | --- | --- | --- | --- | --- | --- | --- | --- | --- |
| **1** | 17 | - | | GastricAspirate | - | Myc. sp / 20 days | Tuberculosisunspecified | *M. tuberculosis* | Standard treatment | Under treatment |
| **2** | 25 | + | | Liquor | - | Myc.sp / 21 days | Miliary tuberculosis | *M. tuberculosis* | Standard treatment | Completed treatment |
| **3** | 16 | - | | Liquor | - | Myc.sp / 18 days | Tuberculosis unspecified | *M. tuberculosis* | Standard treatment | Completed treatment |
| **4** | 54 | - | | Bronchial Aspirate | + | Myc. sp / 15 days | Pulmonary Tuberculosis | *M. tuberculosis* | Standard treatment | Completed treatment |
| **5** | 48 | - | | Sputum | + | Myc. sp / 20 days | Pulmonary Tuberculosis | M. tuberculosis | Standard treatment | Completed treatment |
| **6** | 44 | + | | Liquor | - | Myc. sp / 26 days | Meningitistuberculosis | *M. tuberculosis* | Standard treatment | Completed treatment |
| **7** | 64 | - | | Bronchial Aspirate | + | Myc. sp / 16 days | Pulmonary Tuberculosis | *M. tuberculosis* | Standard treatment | Completed treatment |
| **8** | 27 | - | | Sputum | + | Myc. sp / 21 days | Pulmonary Tuberculosis | *M. tuberculosis* | Standard treatment | Completed treatment |
| **12** | 11 | - | | Bronchial Aspirate | + | Myc. sp / 13 days | Pulmonary Tuberculosis | M. tuberculosis | Standard treatment | Completed treatment |
| **13** | 34 | - | | Bronchial Aspirate | + | Myc. sp / 18 days | Pulmonary Tuberculosis | *M. tuberculosis* | Standard treatment | Completed treatment |
| **15** | 64 | - | | Bronchial Aspirate | + | Myc. sp / 30 days | Pulmonary Tuberculosis | *M. tuberculosis* | Standard treatment | Under treatment |
| **16** | 24 | - | | Bronchial Aspirate | + | Myc. sp / 20 days | Pulmonary Tuberculosis | M. tuberculosis | Standard treatment | Completed treatment |
| **17** | 46 | - | | BronchialAspirate | + | Myc. sp / 40 days | Pulmonary Tuberculosis | *M. tuberculosis* | Standard treatment | Under treatment |
| **18** | 53 | - | | vesicular fluid | + | Myc. sp / 9 days | Vesical tuberculosis | *M. tuberculosis* | Standard treatment | Under treatment |
| **19** | 46 | - | | Bronchial Aspirate | + | Myc. sp / 22 days | PulmonaryTuberculosis | *M. tuberculosis* | Standard treatment | Under treatment |
| **20** | 38 | + | | Cervical Lymph node | - | Myc. sp / 20 days | Pleural tuberculosis | *M. tuberculosis* | Standard treatment | Completed treatment |
| **23** | 28 | - | | tracheal aspirate | + | Myc. sp / 15 days | Pulmonary Tuberculosis | *M. tuberculosis* | Standard treatment | Completed treatment |
| **24** | 65 | - | | Bronchial Aspirate | - | Myc. sp 34 days | Pulmonary Tuberculosis | *M. tuberculosis* | Standard treatment | Completed treatment |
| **26** | 34 | - | | Sputum | + | Myc. sp / 40 days | Pulmonary Tuberculosis | *M. tuberculosis* | Standard treatment | Under treatment |
| **27** | 53 | - | | Liquor | - | Myc. sp / 20 days | Miliarytuberculosis | *M. tuberculosis* | Standard treatment | Under treatment |
| **29** | 30 | + | | Lymph node Aspirate | - | Myc. sp / 15 days | Ganglionar tuberculosis | *M. tuberculosis* | Standard treatment | Under treatment |
| **30** | 33 | - | | Sputum | + | Myc. sp / 15 days | PulmonaryTuberculosis | *M. tuberculosis* | Standard treatment | Under treatment |
| **35** | 27 | - | | tracheal aspirate | - | Myc. sp / 20 days | PulmonaryTuberculosis | *M. tuberculosis* | Standard treatment | Completed treatment |
| **42** | 27 | - | | Sputum | + | Myc. sp / 21 days | PulmonaryTuberculosis | *M. tuberculosis* | Standard treatment | Completed treatment |
| **43** | 27 | + | | Sputum | + | Myc. sp / 12 days | PulmonaryTuberculosis | *M. tuberculosis* | Standard treatment | Under treatment |
| **44** | 54 | - | | Bronchial Aspirate | + | Myc. sp / 10 days | MiliaryTuberculosis | *M. tuberculosis* | Standard treatment | Completed treatment |
| **45** | 66 | - | | Bronchial Aspirate | + | Myc. sp / 20 days | PulmonaryTuberculosis | *M. tuberculosis* | Standard treatment | Completed treatment |
| **46** | 61 | - | | Bronchial Aspirate | + | Myc. sp / 18 days | PulmonaryTuberculosis | *M. tuberculosis* | Standard treatment | Under treatment |
| **47** | 75 | - | | gastric aspirate | - | Myc. sp / 15 days | Pleural tuberculosis | *M. tuberculosis* | Standard treatment | Died |
| **48** | 49 | - | | Bronchial Aspirate | + | Myc. sp / 28 days | PulmonaryTuberculosis | *M. tuberculosis* | Standard treatment | Completed treatment |
| **49** | 54 | - | | Bronchial Aspirate | - | Myc. sp / 25 days | PulmonaryTuberculosis | *M. tuberculosis* | Standard treatment | Completed treatment |
| **50** | 52 | - | | Bronchial Aspirate | - | Myc. sp / 10 days | PulmonaryTuberculosis | *M. tuberculosis* | Standard treatment | Completed treatment |
| **51** | 66 | - | | Sputum | - | Myc. sp / 20 days | PulmonaryTuberculosis | *M. tuberculosis* | Standard treatment | Completed treatment |
| **53** | 61 | - | | gingival biopsy | - | Myc. sp / 20 days | Pleural tuberculosis | *M. tuberculosis* | Standard treatment | Completed treatment |
| **55** | 27 | + | | liver fragment | + | Myc. sp / 20 days | Tuberculosisunspecified | *M. tuberculosis* | Standard treatment | Completed treatment |
|  | | |  | | | | | | | |

Standard treatment for TB (2RHZE / 4RH): two months COCXIP 4 (rifampicin, isoniazid, pyrazinamide and ethambutol) + 4 months ofrifampicin and isoniazid)
